# The NMDA Receptor Promotes Sleep in the Fruit Fly, *Drosophila melanogaster*

**DOI:** 10.1371/journal.pone.0128101

**Published:** 2015-05-29

**Authors:** Jun Tomita, Taro Ueno, Madoka Mitsuyoshi, Shoen Kume, Kazuhiko Kume

**Affiliations:** 1 Institute of Molecular Embryology and Genetics, Kumamoto University, Kumamoto, Japan; 2 Department of Sensory and Motor Systems, Tokyo Metropolitan Institute of Medical Science, Tokyo, Japan; 3 Department of Neuropharmacology, Graduate School of Pharmaceutical Sciences, Nagoya City University, Nagoya, Japan; Universitaet Regensburg, GERMANY

## Abstract

Considerable evidence indicates that sleep is essential for learning and memory. *Drosophila melanogaster* has emerged as a novel model for studying sleep. We previously found a short sleeper mutant, *fumin* (*fmn*), and identified its mutation in the dopamine transporter gene. We reported similarities in the molecular basis of sleep and arousal regulation between mammals and *Drosophila*. In aversive olfactory learning tasks, *fmn* mutants demonstrate defective memory retention, which suggests an association between sleep and memory. In an attempt to discover additional sleep related genes in *Drosophila*, we carried out a microarray analysis comparing mRNA expression in heads of *fmn* and control flies and found that 563 genes are differentially expressed. Next, using the pan-neuronal *Gal4* driver *elav*-*Gal4 and UAS*-RNA interference (RNAi) to knockdown individual genes, we performed a functional screen. We found that knockdown of the NMDA type glutamate receptor channel gene (*Nmdar1*) (also known as *dNR1*) reduced sleep. The NMDA receptor (NMDAR) plays an important role in learning and memory both in *Drosophila* and mammals. The application of the NMDAR antagonist, MK-801, reduced sleep in control flies, but not in *fmn*. These results suggest that NMDAR promotes sleep regulation in *Drosophila*.

## Introduction

Sleep is a physiological state with unique characteristics. Behaviorally, sleep constitutes a consolidated period of rest and immobility without bulky movements, accompanied by an apparent reduced responsiveness to outside stimuli. The amount of sleep is regulated by both circadian and homeostatic processes [1]. Scientifically, sleep has been defined by electroencephalogram criteria in humans [2] and thus is mainly described in mammalian and avian species. In insects, sleep-like resting states were described first in the cockroach [3] and in the fruit fly, *Drosophila melanogaster* [4, 5]. We discovered a mutant with a reduced amount of these sleep-like states, and named it *fumin*, meaning 'insomnia' in Japanese and identified the mutation in the dopamine transporter gene [6]. This was a striking finding, since dopamine is also used to maintain wakefulness in the mammalian brain. Thus, it demonstrated the similarity between the molecular mechanism for regulating sleep-like states in *Drosophila* to that of the mammalian system [6]. Since then, cumulative evidence has continued to reveal similarities with mammalian sleep, so the sleep-like state of *Drosophila* is now simply called “*Drosophila* sleep”.

In addition to similar behavioral characteristics and molecular mechanisms, mammalian sleep and *Drosophila* sleep share common physiological traits. Many short sleeping mutants have a reduced life span [7–10], but despite their short sleeper phenotype, *fmn* have an equivalent life span to control flies [6]. In addition, together with other short sleep mutants, *fmn* have a memory retention defect and deprivation of sleep impairs their memory [11]. These findings indicate that sleep plays an important role in lifespan and memory in *Drosophila* and may provide an insight into why sleep evolves in a broad range of species.

In order to further elucidate the molecular mechanisms regulating sleep in *Drosophila*, we attempted to isolate more sleep related genes using the *fmn* mutant. In this report, we describe a gene expression analysis of *fmn*, followed by the successful isolation of the *N*-methyl-d-aspartic acid type glutamate receptor channel (NMDAR) gene, *Nmdar1*, as a novel sleep related gene and its function in promoting sleep.

## Materials and Methods

### Fly strains and culture conditions

Flies were reared at 25°C in 50–60% relative humidity on standard fly food consisting of corn meal, yeast, glucose, wheat germ and agar. They were kept under 12 h light (zeitgeber time, ZT 0–12) followed by 12 h dark (ZT 12–24) cycles defined as the LD conditions. The transgenic RNA interference (RNAi) lines for *Nmdar1* (VDRC 37333 and 104773), *UAS*-*Dicer*-*2* flies (60008) and the *w* ^*1118*^ (60000) which is the genetic background of the RNAi line were obtained from the Vienna *Drosophila* RNAi Center (VDRC) [12]. MB247 was provided by Dr. Hiromu Tanimoto. 11Y, 30Y, 201Y, 7Y, 104Y, 121Y, c232 and c767 were a gift from Dr. J. Douglas Armstrong. c747 and c772 were provided by Dr. Toshiro Aigaki. *TH*-*Gal4* was a gift from Dr. Jay Hirsh. *GAD*-*Gal4* was provided by Dr. Takaomi Sakai. OK371-*Gal4* was a gift from Dr. Hermann Aberle. *dilp2*-*Gal4* was a gift from Dr. Linda Partridge. *npf*-*Gal4* was a gift from Dr. Ping Shen. OK307-*Gal4* and c17 were a gift from Dr. Tanja Godenschwege. *tsh*-*Gal4* was provided by Dr. Julie H. Simpson. The following stocks were ordered from the Bloomington Stock Center, Indiana University: *elav*-*Gal4* (Stock number: 458), c309 (6906), c739 (7362), *Ddc*-*Gal4* (7009), *Tdc2*-*Gal4* (9313), *Cha*-*Gal4* (6793), *dimm*-*Gal4* (25373), *per*-*Gal4* (7127), *tim*-*Gal4* (7126), *repo*-*Gal4* (7415) and D42 (8816). NP3529, NP6510, NP10, NP1004 and NP5103 were obtained from the *Drosophila* Genetics Resource Center, Kyoto Institute of Technology, Japan. The dopamine transporter mutant *fumin* (*fmn*) flies were isolated in our laboratory in a stock of *y w* flies, and backcrossed over five generations to the control strain (*w* ^*1118*^) [6]. The backcrossed *w*; *fmn* line and the original *y w*; *fmn* line showed similar short sleeping phenotypes. Knockdown and control flies were obtained by crossing the pan-neuronal *Gal4* driver *elav*-*Gal4* with the *UAS*-*Dicer*-*2* on the second chromosome to each of the RNAi lines and *w* ^*1118*^, respectively. The 2- to 4-d-old post-eclosion male flies were used in this study.

### Microarray analysis

Control (*w* ^*1118*^) and *fmn* flies subjected to four LD cycles were harvested every 4 h at seven time points under the following LD conditions (ZT 0, 4, 8, 12, 16, 20 and 24) and immediately frozen in liquid nitrogen. Frozen flies were vigorously shaken and sieved to collect the heads which had separated from the bodies. Total RNA was extracted from approximately 400 male and female fly heads homogenized in TRIzol reagent (Invitrogen) and its quality was assessed with an RNA 600 Nano Assay Kit using the Agilent 2100 Bioanalyzer (Agilent Technologies). Double-stranded cDNA was synthesized from 5 μg of total RNA using a One-Cycle cDNA Synthesis Kit (Affymetrix) and served as a template to synthesize biotin-labeled cRNA using a GeneChip IVT Labeling Kit (Affymetrix). Biotin-labeled cRNA was fragmented and hybridized to an Affymetrix GeneChip *Drosophila* Genome 2.0 Array, which represents the entire *Drosophila* genome with over 18,500 transcripts, as recommended by the manufacturer. Hybridized arrays were washed and stained using a GeneChip Hybridization, Wash and Stain Kit (Affymetrix) on a Fluidics Station 400, and scanned on an Affymetrix GeneChip Scanner 3000. Expression measures for each probe set were calculated using the MAS5 algorithm. To find differentially expressed genes in control and *fmn* fly heads, data were normalized, prefiltered and analyzed by the Subio Platform (http://www.subio.jp/). Probe sets that were not called Present by the MAS5 and whose raw signal intensity were less than 100 in at least 7 of 14 samples were excluded from analysis. We identified the 8,740 probe sets above the threshold. Subsequently, a *t*-test was performed on the prefiltered list. The Benjamini-Hochberg false discovery rate (FDR) was set to 5%. At a cutoff of at least 1.25 × up or down, 563 genes were determined to be differentially expressed between control and *fmn* flies. Functional Annotation Chart tool in the Database for Annotation, Visualization and Integrated Discovery (DAVID) 6.7 was used to find enriched gene ontology (GO) terms in the biological process category. Count threshold, minimum number of differentially expressed genes involved in individual term was set to 10. The *p*-value was set to < 0.05.

### mRNA analysis by quantitative RT-PCR

The efficiency and specificity of *Nmdar1* RNAi were examined by quantitative RT-PCR (qPCR). For biological replicates, the knocked down and control male flies were collected from each of three culture vials at ZT 6. Total RNA was prepared from 35–40 heads by using TRIzol reagent (Invitrogen) according to the manufacturer’s instructions. cDNA synthesized from the total RNA using oligo (dT)_20_ primer and ReverTra Ace reverse transcriptase (Toyobo) was used for qPCR using THUNDERBIRD SYBR qPCR Mix (Toyobo). *GAPDH2* expression levels were quantified and used as an internal control. The primers were 5ʹ-AGGAAGGAAA AGCGGAAAAG-3ʹ and 5ʹ-GGGGAGGATA AACGAGGTGT-3ʹ for *Nmdar1*, 5ʹ-TCGGTTCGGT TTGGATGAG-3ʹ and 5ʹ-TTGTCCTTTC CGCCTGTATG T-3ʹ for *GluRIB*, 5ʹ-CCCCCAAAAT GGAAGCTAAT-3ʹ and 5ʹ-TGGCAACTGC TTCGTGTCTA-3ʹ for *CanA*-*14F*, 5ʹ-TGCTGTCGAG CGAGTAGAGA-3ʹ and 5ʹ-ATGCTGGCCT TTGGTTACTT-3ʹ for *CanB*, 5ʹ-GAAGAAGCGC ACCAAGCACT-3ʹ and 5ʹ-TTGAATCCGGTGGGCAGCAT-3ʹ for *RpL32* (*rp49*) and 5ʹ-TGGTACGACA ACGAGTTTGG-3ʹ and 5ʹ-TTTCAGGCCG TTTCTGAAGT-3ʹ for *GAPDH2*. First, each mRNA expression level was normalized to *GAPDH2*. Then, the normalized values were normalized to the average of three independent control samples, which was set at 100%.

### Locomotor activity and sleep analysis

Flies were individually housed in glass tubes (length, 65 mm; inside diameter, 3 mm) containing either standard fly food or sucrose-agar (1% agar supplemented with 5% sucrose) at one end and were entrained for at least 3 d under LD cycle conditions before being transferred to constant dark (DD) conditions. Locomotor activity was monitored by recording the number of infrared beam crossings for individual flies in 1 min bins using the *Drosophila* activity monitoring (DAM) system (Trikinetics). Data were continuously collected for 3 d under either LD or DD conditions. Under LD, ZT was used and under DD, circadian time (CT, with CT 0 as 12 h after lights-off of the last LD conditions) was used to indicate daily time, respectively. Based on previous reports [4–6, 13], sleep was defined as periods of inactivity lasting 5 min or longer. Total activity, total sleep, sleep bout length and sleep bout number in LD and DD conditions were analyzed by a Microsoft Excel-based software as previously described [6] and averaged over 3 d for each condition. This software was used to scan activity data over 3 d in 1 min bins, i.e., a total of 4,320 bins. Five or more continuous bins with the value of zero (lack of activity) are regarded as one sleep bout and the bins composing sleep bouts were assigned to sleep. To quantify sleep, the number of bins assigned to sleep were counted and expressed as a percentage of the total bins. The number and the length of each sleep bout were also recorded and the mean bout length of sleep was calculated. For the pharmacological experiments, sucrose-agar food was used as a control. (+)-MK-801 hydrogen maleate (MK-801) and 3-iodo-L-tyrosine (3IY) were purchased from Sigma-Aldrich. MK-801 or/and 3IY was directly mixed with sucrose-agar food at a concentration of 0.1 mg/ml and 3 mM, respectively.

### Statistical analysis

Data were analyzed as described in the figure legends using Microsoft Excel and the freely available statistical software package R 3.0.0. (http://www.r-project.org/).

## Results

### Microarray analysis

**Fig 1:**
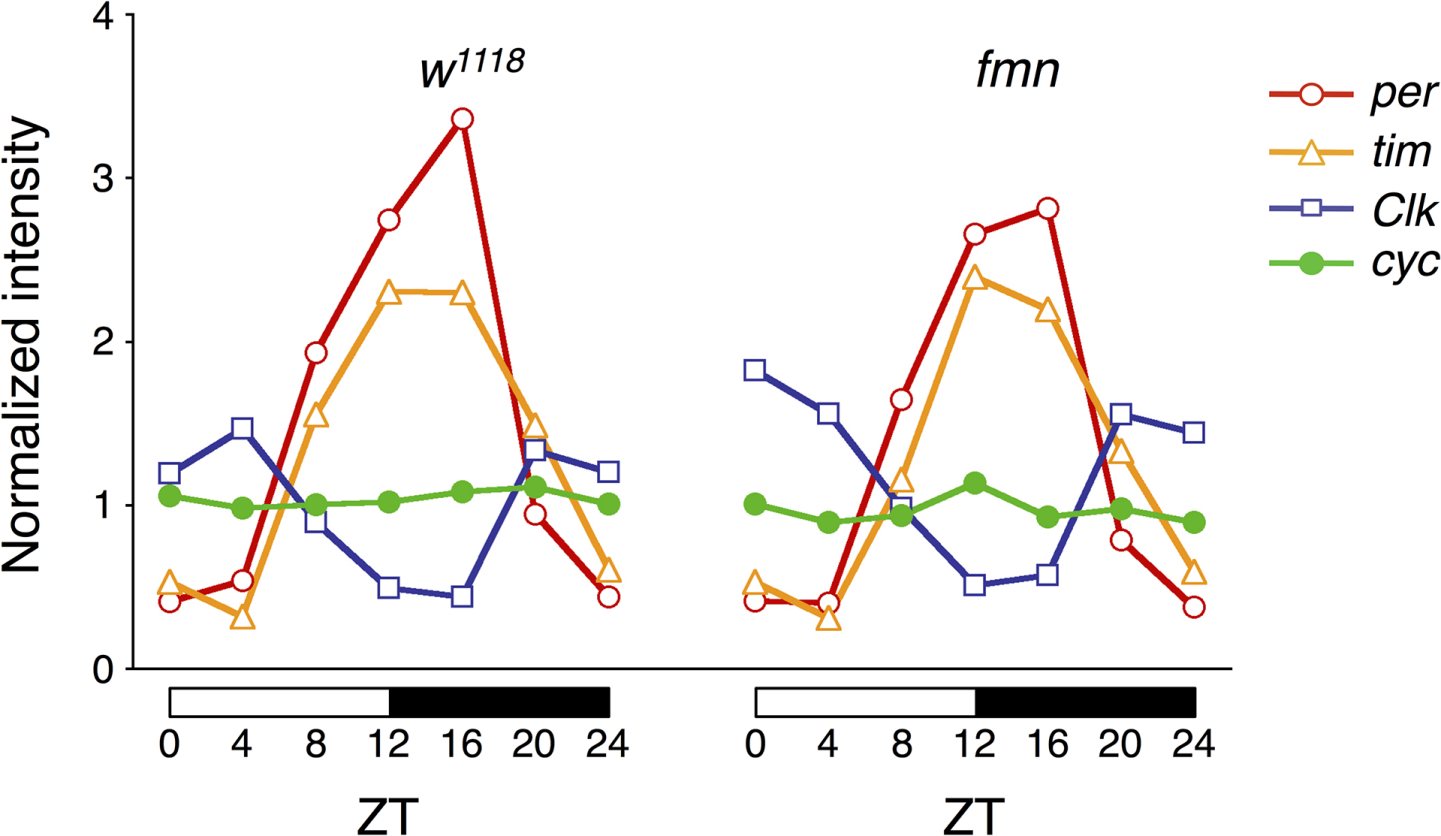
Clock gene cycling is not altered in *fmn*. Temporal expression patterns of circadian clock genes (*per*, *tim*, *Clk* and *cyc*) in the heads of control (*w* ^*1118*^) and *fmn* flies under LD conditions. Data were normalized to the average signal intensity from each time point, which were set to 1.

We took RNA samples from the heads of both the control and *fmn* mutant flies which had been backcrossed for 5 generations to the control background flies [6]. Under LD conditions, 7 RNA sample sets were collected every 4 hours around the clock (ZT 0, 4, 8, 12, 16, 20 and 24). The samples at ZT 0 and ZT 24 were overlapped and used as a measure for the experimental variations. The RNAs were analyzed as described in Materials and Methods and the results were uploaded to the Gene Expression Omnibus (GEO) as GSE56149. As shown in Fig 1, circadian clock genes (*per*, *tim*, *Clk*) cycled similarly in both flies and there was no apparent difference between them. The expression level of three dopamine receptor genes (*DopR*, *DopR2*, *D2R*) did not differ in *fmn*. *Tyrosine hydroxylase* and other dopamine related genes including, *Dopamine N acetyltransferase* (*Dat*), L-3,4-dihydroxyphenylalanine (*DOPA*) *decarboxylase* (*Ddc*) and *β*-*alanyl*-*dopamine synthase* (*ebony*) also did not show any difference, suggesting that no compensatory changes in the expression level occurred in *fmn* (data not shown).

Since the *fmn* mutation does not affect circadian rhythm, we statistically compared 7 samples as a whole using a *t*-test with an FDR of 5%, and found that 563 genes showed a significantly different expression level between *fmn* and the control, as shown in S1 Table. Of these, expression levels in 272 genes were up-regulated and 291 genes were down-regulated in *fmn*. Using gene ontology enrichment analysis, we found that the differentially expressed genes in *fmn* are involved in many different biological processes including metabolism, transport, development, protein modification and more (S2 Table). *Dopamine transporter* (*DAT*) gene expression was barely detectable in *fmn*, which validated the experiment (S1 Table). We first noticed the *scab* (*scb*) gene, that codes for α-integrin, and its mutant, called *Volado*, which shows abnormal synaptic transmission and memory deficit [14, 15] and is dramatically down regulated in *fmn*. Thus, we confirmed its expression in the control and *fmn* using RT-PCR. To our disappointment, there was no difference in *scb* mRNA levels between the control and *fmn* when we used primers for the reading frame. By sequencing the *scb* gene of *fmn*, we identified single-base substitutions in the 3' untranslated region, which is on position 1629803_a_at of the Affymetrix DNA array probe set (data not shown), resulting in a very low signal on a microarray. Since the *scb* gene is located on the right arm of the second chromosome (2R) at 51E10-51E11 (Sequence location, 2R: 11,136,290..11,146,003) and *fmn* (*DAT*) at 51E10-51E11 (12,446,062..12,452,763), the distance between the two is 1.3 M bases. Our backcross was apparently insufficient to isogenize this region.

### Functional screening for a sleep related phenotype

**Fig 2:**
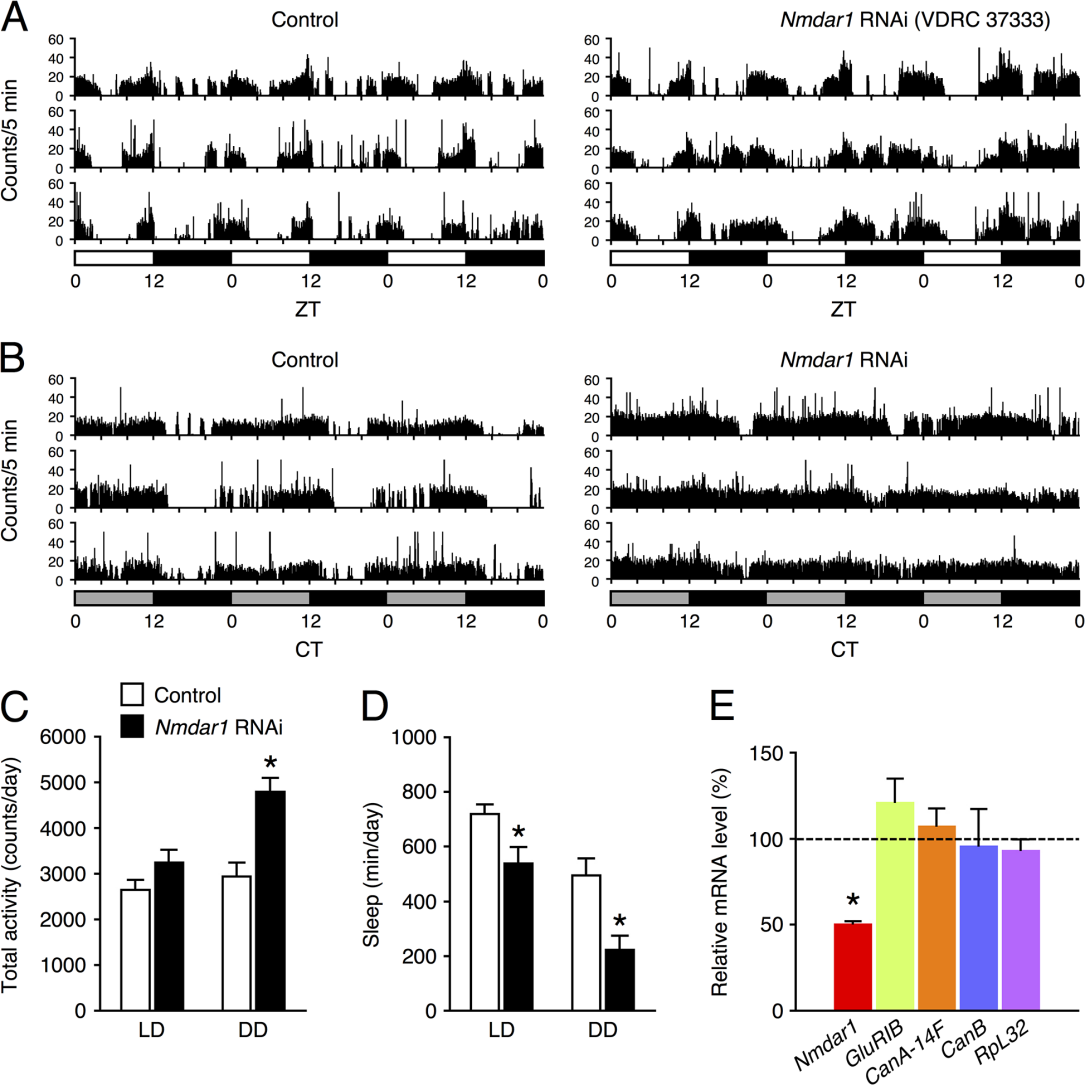
Pan-neuronal *Nmdar1* knockdown decreases sleep. (A, B) Locomotor activity profiles for three different control (*elav*-*Gal4*;*UAS*-*Dicer*-*2* × *w* ^*1118*^, left panel) and *Nmdar1* RNAi (VDRC 37333)-expressing flies using the *elav*-*Gal4*;*UAS*-*Dicer*-*2* driver (right panel) for 3 d in LD (A) and DD conditions (B). White, gray and black bars under the horizontal axis indicate day (ZT 0–12), subjective day (CT 0–12) and night (ZT 12–24 or CT 12–24), respectively. Total daily activity (C) and total sleep (D) for control (white bars) and *Nmdar1* RNAi flies (black bars) in LD and DD conditions. *n* = 16 for each group. Data are presented as mean ± SEM. Asterisks indicate statistically significant differences compared to control according to a *t*-test (*p* < 0.05). (E) Efficiency and specificity of *Nmdar1* gene knockdown. The expression levels of *Nmdar1*, *GluRIB*, *CanA*-*14F*, *CanB* and *RpL32* genes in the head of male flies expressing *Nmdar1* RNAi transgenes in all neurons are expressed as relative values to the control flies. As described in the Materials and Methods, each mRNA level was quantified by qPCR and first normalized to *GAPDH2*. Then, the values were normalized to the average of independent control samples, which were set at 100%. Data are presented as mean ± SEM. *n* = 3 for each group. Statistical significance between control and RNAi flies: *p < 0.05, *t*-test.

Next, we performed a loss-of-function screen to isolate sleep-related genes. As a first trial round of screening, we selected 26 candidate genes with different properties, i.e. genes whose expression levels are largely different but whose functions are unknown and genes which functions in the nervous system, such as receptors for neurotransmitters and molecules in the signal transduction. We knocked these 26 genes and *DAT* down using the pan-neuronal *Gal4* driver, *elav*-*Gal4* in conjunction with *UAS*-RNAi lines [12]. We confirmed that knockdown of *DAT* reduced sleep just like *fmn*, confirming the validity of this method (data not shown). Of the 26 genes, knockdown of three genes, namely *Nmdar1*, *CanA*-*14F* and *CanB* which encode the NMDAR subunit, calcineurin catalytic subunit A and calcineurin regulatory subunit B, respectively, resulted in a significant reduction in sleep. In microarray analysis, fold change (control/*fmn* signal ratio) for *Nmdar1*, *CanA*-*14F* and *CanB* were 1.32, 1.43 and 1.25, respectively (S1 Table). Detailed analysis of the *calcineurin* genes in sleep regulation have been previously reported [16]. Two independent *Nmdar1* RNAi fly lines (VDRC 37333 and 104773) were crossed with *elav*-*Gal4*;*UAS*-*Dicer*-*2* flies. These *Nmdar1* RNAi expressing fly lines were viable and showed no apparent morphological abnormality. Fig 2A and 2B show the activity plot for three different control flies and *Nmdar1* RNAi (VDRC 37333)-expressing flies in LD and DD conditions. The *Nmdar1* knockdown flies displayed significant hyperactivity compared with control flies under DD (Fig 2A–2C) conditions. In contrast, the total daily sleep of the *Nmdar1* RNAi flies was significantly decreased in both LD and DD conditions relative to the control (Fig 2D). We confirmed the efficiency and specificity of the *Nmdar1* knockdown by quantification of mRNA. As shown in Fig 2E, the *Nmdar1* mRNA levels were significantly decreased to approximately 50%. Although the *glutamate receptor IB* (*GluRIB*) gene is reported as a potential off-target gene for the *Nmdar1* RNAi construct by target predictions of the VDRC [12], the *GluRIB* mRNA levels were comparable in the *Nmdar1* RNAi and control flies. We also examined the mRNA levels of *CanA*-*14F* and *CanB* for neuronal genes and *RpL32* for ubiquitously expressed genes and found there were no differences in their mRNA levels in the *Nmdar1* knockdown flies. Another *Nmdar1* RNAi line (104773) gave a significant increase in locomotor activity and reduction in sleep only under DD conditions (S1A and S1B Fig). The *Nmdar1* mRNA levels tended to be reduced in the RNAi flies, but did not reach statistical significance (S1C Fig).

**Fig 3:**
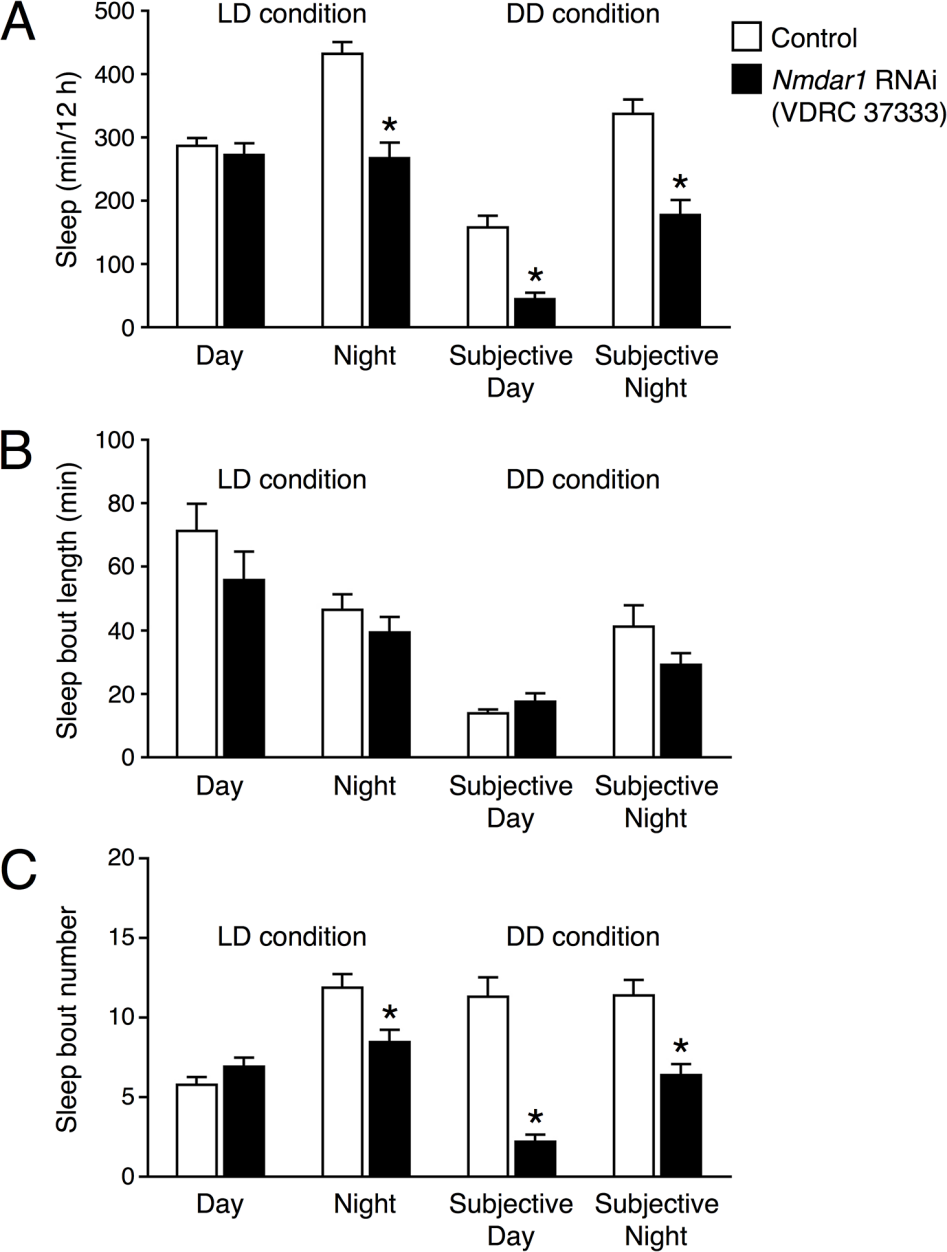
*Nmdar1* knockdown flies show a decreased sleep bout number. Total sleep (A), sleep bout length (B) and sleep bout number (C) for control (*elav*-*Gal4*;*UAS*-*Dicer*-*2* × *w* ^*1118*^, white bars) and *Nmdar1* RNAi (VDRC 37333)-expressing flies using the *elav*-*Gal4*;*UAS*-*Dicer*-*2* (black bars) during day (ZT 0–12), night (ZT 12–24), subjective day (CT 0–12) and subjective night (CT 12–24). A total of 48 flies were tested for both genotypes in (A) and (C). Flies with no sleep during 12 h were excluded from the calculations of sleep-bout length in (B) (*n* = 24–48). Data are presented as mean ± SEM. Asterisks indicate statistically significant differences from control determined by a *t*-test (*p* < 0.05).

Since *Nmdar1* knockdown flies were more active than control flies, especially during the subjective night period under DD conditions, we calculated the parameters of day sleep and night sleep separately. In *Nmdar1* RNAi (37333)-expressing flies, there was no significant difference in the amount of sleep during daytime under LD conditions (Fig 3A). Night sleep under LD conditions and both subjective day and subjective night sleep under DD conditions were significantly shorter in the *Nmdar1* knockdown flies. There were no significant differences in the bout length of sleep in the *Nmdar1* RNAi and control flies under both LD and DD conditions (Fig 3B). As shown in Fig 3C, the decrease in sleep in the *Nmdar1* RNAi flies was mainly due to the reduction in sleep bout number. Similarly, another *Nmdar1* RNAi line (104773) gave a significant decrease not in sleep bout length but in sleep bout number (S2B and S2C Fig).

### An NMDAR antagonist reduces sleep

**Fig 4:**
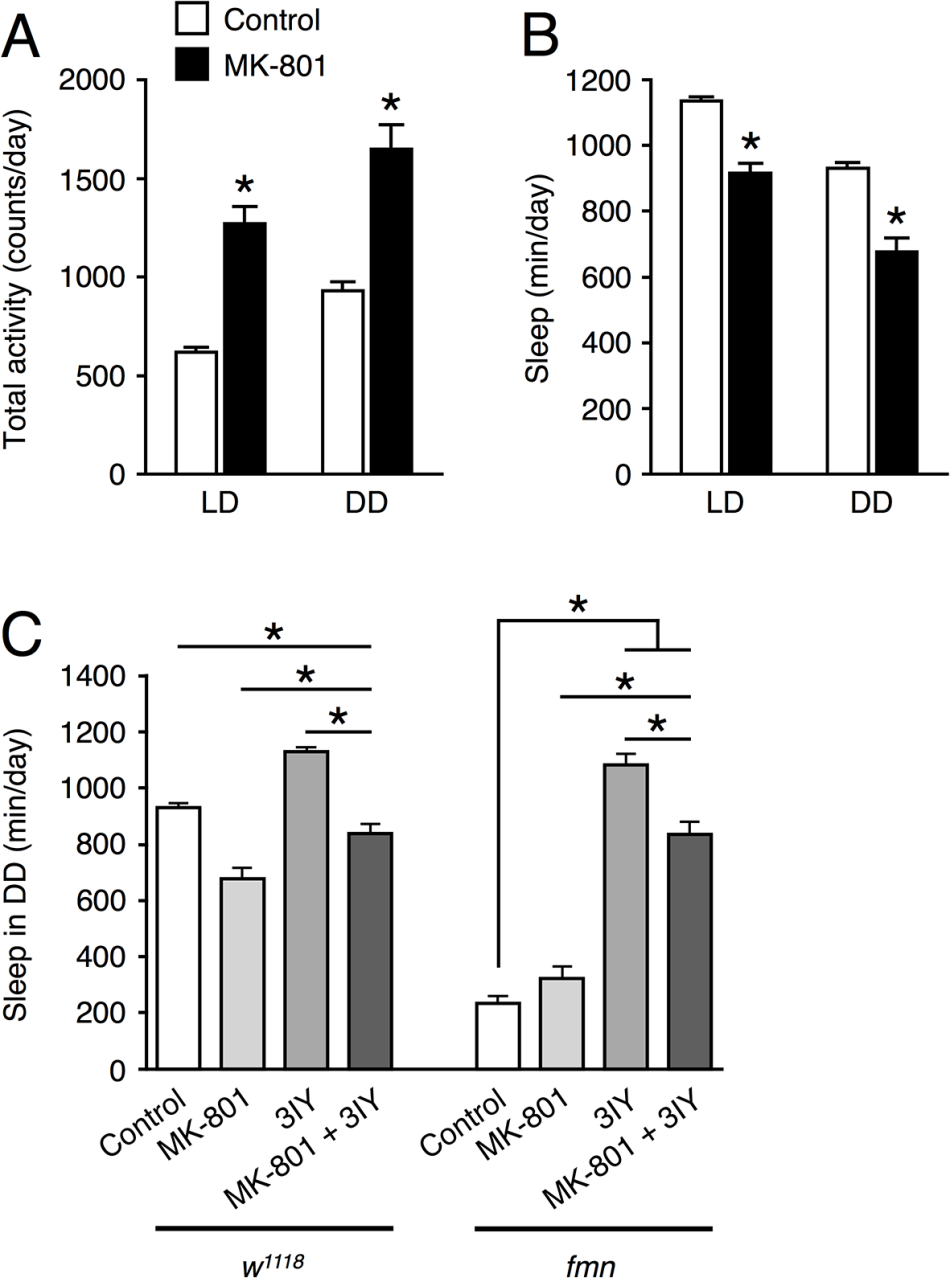
The NMDAR antagonist MK-801 reduces sleep. Total daily activity (A) and total sleep (B) for untreated control (white bars, *n* = 32) and MK-801-fed *w* ^*1118*^ flies (black bars, *n* = 28) in LD and DD conditions. Data are presented as mean ± SEM. Asterisks indicate statistically significant differences compared to control according to a *t*-test (*p* < 0.05). (C) Effect of administration of MK-801 and tyrosine hydroxylase inhibitor, 3-iodo-L-tyrosine (3IY) on sleep in *w* ^*1118*^, the genetic background of *fmn* mutants, and *fmn* flies in DD. MK-801 or/and 3IY was directly mixed with sucrose-agar food at a concentration of 0.1 mg/ml and 3 mM, respectively. Data are presented as mean ± SEM (*n* = 28–32 for each group). Groups with asterisks indicate statistically significant differences (Tukey-Kramer HSD test for normally distributed data, *p* < 0.05).

**Fig 5:**
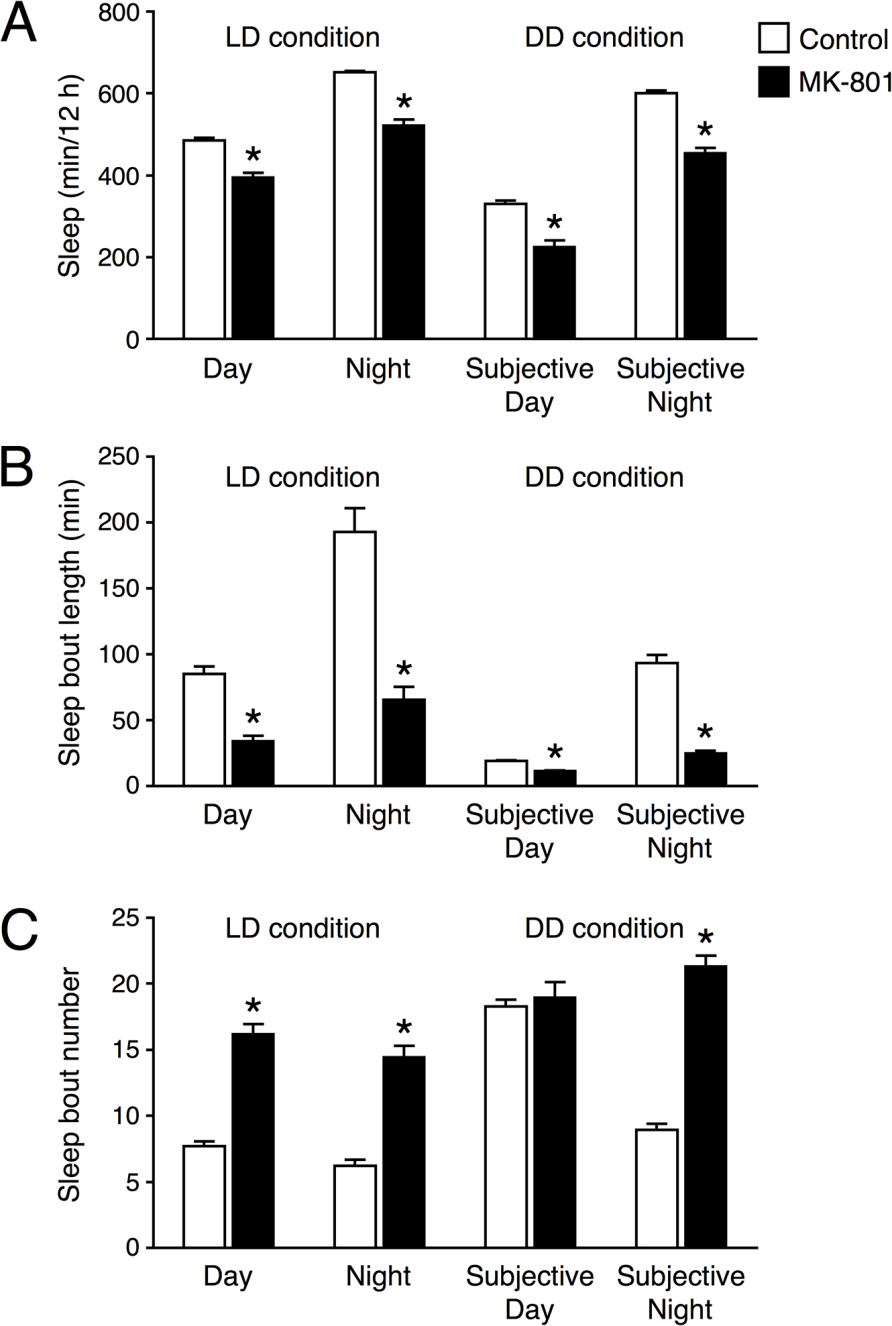
MK-801 reduces sleep bout length. Total sleep (A), sleep bout length (B) and sleep bout number (C) for untreated control (white bars, *n* = 96) and MK-801-treated *w* ^*1118*^ flies (black bars, *n* = 84) during day (ZT 0–12), night (ZT 12–24), subjective day (CT 0–12) and subjective night (CT 12–24). Data are presented as mean ± SEM. Asterisks indicate statistically significant differences from control determined by a *t*-test (*p* < 0.05).

In order to pharmacologically examine NMDAR function on sleep, we assessed the effect of the NMDAR antagonist, MK-801. MK-801 is a non-competitive antagonist, which binds to the phencyclidine binding site of NMDAR and prevents its channel activities. We mixed MK-801 into the food and fed the flies during the sleep assay. At concentrations of 0.1 mg/ml, amount of sleep was significantly decreased compared to untreated controls, whereas lower doses (0.01 and 0.03 mg/ml) had no effect on sleep (S3 Fig). In 16 flies with 0.1 mg/ml MK-801, 3 ones died during the sleep assay. Thus, we used 0.1 mg/ml MK-801 in following experiments. As shown in Fig 4, MK-801 reduced sleep significantly in *w* ^*1118*^ flies but not in *fmn* flies, which suggested that the effect of the *Nmdar1* knockdown was due to a nondevelopmental effect. Although the *Nmdar1* RNAi flies showed the reduction in sleep bout number, administration of MK-801 shortened sleep bout length (Fig 5B). We also examined the effects of the tyrosine hydroxylase inhibitor, 3-iodo-L-tyrosine (3IY), which inhibits dopamine biosynthesis. 3IY increased the amount of sleep in *fmn* almost to the control level (Fig 4C). Simultaneous addition of 3IY with MK-801, partially hindered the effects of MK-801, suggesting the additive effects of MK-801 and dopamine signaling in reducing sleep. These data indicated that NMDAR functions to promote sleep in *Drosophila*.

### *Nmdar1* knockdown in various regions of the fly brain

**Fig 6:**
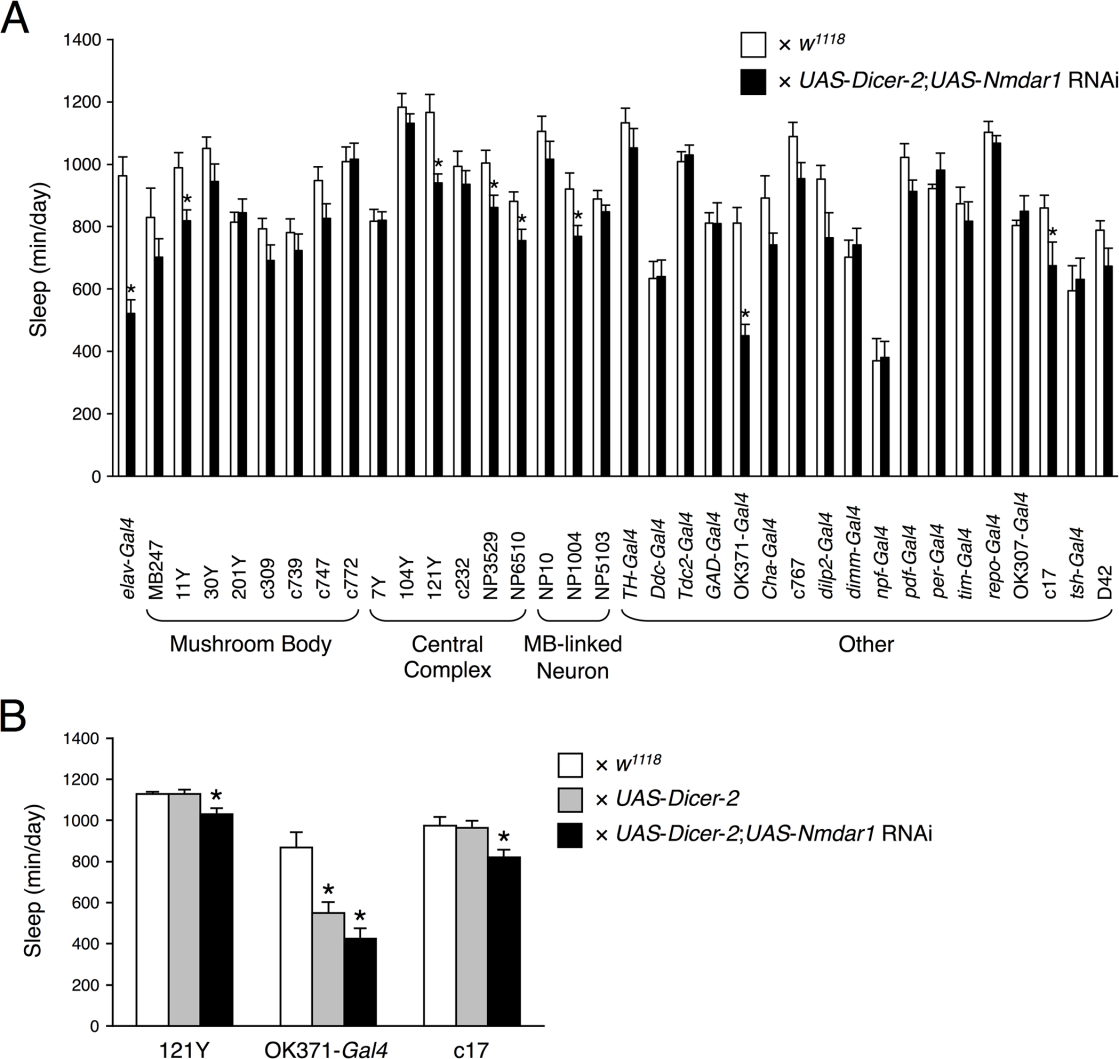
*Nmdar1* knockdown in various regions of the fly brain. (A) Total sleep for progeny collected from between 36 *Gal4* drivers and *UAS*-*Nmdar1* RNAi (VDRC 37333) with the *UAS*-*Dicer*-*2* on the 2nd chromosome in DD conditions (black bars). For controls, each *Gal4* driver was crossed to *w* ^*1118*^ flies (white bars). Data are presented as mean ± SEM (*n* = 6–8 for each group). Asterisks indicate statistically significant differences from control determined by a *t*-test (*p* < 0.05). (B) Total sleep for flies expressing *Nmdar1* RNAi and *Dicer*-*2* transgenes under indicated *Gal4* drivers (black bars) in DD conditions. For controls, each *Gal4* driver was crossed to *w* ^*1118*^ (white bars) or *UAS*-*Dicer*-*2* flies (gray bars). Data are presented as mean ± SEM (*n* = 7–16 for each group). Groups with asterisks indicate statistically significant differences (Tukey-Kramer HSD test for normally distributed data, *p* < 0.05).

To identify neurons important for the function of NMDAR in sleep regulation, we screened 36 neural *Gal4* drivers for their potency to induce *Nmdar1* knockdown-induced decreased sleep (Fig 6A). *Nmdar1* knockdown in the mushroom body or central complex did not show the reduced sleep comparable to that in the pan-neuronal knockdown flies. *Nmdar1* knockdown in the fan-shaped body (104Y), which receives projection of sleep-regulating dopaminergic neurons [17, 18] also did not decrease sleep. On the other hand, *Nmdar1* knockdown by at least two *Gal4* drivers (121Y and c17) significantly decreased sleep (Fig 6B), suggesting that NMDAR expressed in several brain regions were involved in sleep regulation.

## Discussion

This study provides evidence that NMDAR is involved in the regulation of sleep in flies.

To identify molecular candidates involved in sleep and arousal, we performed a genome-wide screen of genes that were differentially expressed in heads of control and *fmn* flies. Since DAT functions as part of the clearance mechanism for released dopamine, extracellular dopamine levels are persistently elevated and postsynaptic dopamine signaling is increased in the *DAT* mutant *fmn*. Although we predicted that expression of dopamine-related genes including dopamine receptors and genes of the dopamine biosynthesis pathway would be altered in *fmn* flies, such adaptive changes were not observed at the mRNA level. In *DAT* knockout mice, the levels of both D1 and D2 dopamine receptors are significantly reduced [19]. We ourselves found that D1 dopamine receptor protein level was reduced in the mushroom body area of *fmn* mutant [20]. Therefore, it is possible that expression levels of these genes are posttranscriptionally regulated in *fmn* flies. Oscillation of circadian clock genes, *per*, *tim* and *Clk* under LD conditions were barely affected by the *fmn* mutation (Fig 1) and *fmn* flies showed a substantial circadian rhythmicity of locomotor activity [6], suggesting that *fmn* flies have intact molecular clocks. We identified 563 genes with a significant difference in expression levels over a day between control and *fmn* flies using microarray analysis (at 5% FDR). Of the 563 genes, 272 genes were up-regulated and the remaining 291 genes including *DAT* were down-regulated in *fmn* flies. Although the *scb* gene was identified as a markedly down-regulated gene in *fmn* flies in the microarray data, the false difference in expression was due to a single-base substitution in the 3ʹ untranslated region, for which probe set was designed. This is a problem of gene expression analysis using this type of DNA microarray. Furthermore, it should be noted that our backcross was apparently insufficient to isogenize a neighboring *scb* locus. Gene ontology enrichment analysis using the 563 differentially expressed genes suggested a number of altered biological processes in *fmn* flies, listed in S2 Table. The changes in expression detected in many genes involved in metabolism likely reflect hyperactivity in locomotion in *fmn* flies [6] and dopamine function in modulating metabolic rate [21]. Interestingly, genes involved in response to stress are also identified as wakefulness-related genes in *Drosophila* and rats [22, 23]. We found that differentially expressed genes were involved in nervous system development. These findings may be associated with the physiological function of sleep and regulation of neuronal plasticity in *Drosophila* [24, 25]. Using transgenic RNAi screening, we showed that a pan-neuronal knockdown of *Nmdar1* resulted in locomotor hyperactivity and sleep reduction (Fig 2 and S1 Fig). Moreover, the application of an NMDAR blocker, MK-801 also reduced sleep in control flies (Fig 4). These results indicate a sleep-promoting role for NMDAR in *Drosophila*. We observed that *Nmdar1* knockdown decreased sleep bout number, while MK-801 shortened sleep bout length (Fig 3, S2 Fig and Fig 5). These different effects may be due to the difference in the degree of inhibition of NMDAR function, or it is possible the pharmacological interference by NMDAR antagonist may not mimic NMDAR knockdown. Sleep was unaffected in *fmn* flies that were treated with MK-801 (Fig 4). This can be explained by the decrease in *Nmdar1* gene expression in *fmn*. As previously reported, tyrosine hydroxylase inhibitor 3IY-treated *fmn* flies demonstrated significantly increased sleep levels, almost similar to that of control flies [26]. Simultaneous administration of 3IY and MK-801 partially suppressed the effects of MK-801 in controls and those of 3IY in *fmn* flies, suggesting an additive effect for MK-801 and dopamine signaling in reducing sleep. Although we have no data on the physiological relationship between NMDAR and dopamine signaling in sleep regulation, calcineurin, a heterodimeric Ca^2+^/calmodulin-dependent serine/threonine protein phosphatase, may link these two pathways. We found that *CanA*-*14F* and *CanB* genes were down-regulated in *fmn* flies (S1 Table). Calcineurin is activated by Ca^2+^ influx through the NMDAR. We recently reported that pan-neuronal *CanA*-*14F* and *CanB* RNAi flies significantly decrease sleep in a similar way to *Nmdar1* RNAi flies [16]. In the mammalian brain, calcineurin dephosphorylates dopamine- and cAMP-regulated phosphoprotein (DARPP-32) and thus modulates neuronal functions [27–29]. The *Drosophila* NMDAR subunits, Nmdar1 (dNR1) and Nmdar2 (dNR2), are widely expressed in the adult brain [30–33]. NMDAR expressed in the mushroom body and central complex is required for memory formation [31, 32]. The mushroom body has been shown to function not only as a memory center but also as a sleep center in *Drosophila* [34–36]. However, *Nmdar1* knockdown in these brain regions hardly decreased sleep (Fig 6). The physiological association between sleep and memory in *Drosophila* has recently been described in various reports. Sleep deprivation causes impairment in aversive olfactory learning and memory [37, 38]. The short sleeper *fmn* mutants have poor memory retention [11]. Another short sleep mutant, *Hyperkinetic* [39] and *CanA*-*14F* RNAi flies [16] also have an impaired memory. Taken together, our results indicate that NMDAR-calcineurin signaling plays an important role not only in memory function but also in sleep regulation. Understanding NMDAR-calcineurin signaling in *Drosophila* may provide novel insights into the molecular relationship between sleep and memory.

## Supporting Information

S1 Fig Pan-neuronal *Nmdar1* knockdown decreases sleep. Total daily activity (A) and total sleep (B) for control (*elav*-*Gal4*;*UAS*-*Dicer*-*2* × *w* ^*1118*^, white bars, *n* = 19) and *Nmdar1* RNAi (VDRC 104773)-expressing flies using the *elav*-*Gal4*;*UAS*-*Dicer*-*2* driver (black bars, *n* = 20) in LD and DD conditions. Data are presented as mean ± SEM. Asterisks indicate statistically significant differences compared to control according to a *t*-test (*p* < 0.05). (C) Efficiency of *Nmdar1* gene knockdown. The expression levels of *Nmdar1* and *RpL32* genes in the head of male flies expressing *Nmdar1* RNAi transgenes in all neurons are expressed as relative values to the control flies. Each mRNA level was quantified by qPCR and first normalized to *GAPDH2*. Then, the values were normalized to the average of independent control samples, which were set at 100%. Data are presented as mean ± SEM. *n* = 3 for each group.
(PDF)

S2 Fig *Nmdar1* knockdown flies show a decreased sleep bout number. Total sleep (A), sleep bout length (B), and sleep bout number (C) for control (*elav*-*Gal4*;*UAS*-*Dicer*-*2* × *w* ^*1118*^, white bars, *n* = 57) and *Nmdar1* RNAi (VDRC 104773)-expressing flies using the *elav*-*Gal4*;*UAS*-*Dicer*-*2* (black bars, *n* = 60) during day (ZT 0–12), night (ZT 12–24), subjective day (CT 0–12) and subjective night (CT 12–24). Data are presented as mean ± SEM. Asterisks indicate statistically significant differences from control determined by a *t*-test (*p* < 0.05).
(PDF)

S3 Fig Addition of the NMDAR antagonist MK-801 to the food at a concentration of 0.1 mg/ml decreases sleep. Total sleep for untreated control (white bars) and MK-801-fed *w* ^*1118*^ flies (colored bars) in DD conditions. MK-801 was directly mixed with sucrose-agar food at indicated concentration. Data are presented as mean ± SEM (*n* = 13–16 for each group). Groups with asterisks indicate statistically significant differences (Tukey-Kramer HSD test for normally distributed data, *p* < 0.05).
(PDF)

S1 Table List of all genes that were significantly differentially expressed between control (*w* ^*1118*^) and *fmn* flies (*t*-test, 5% FDR; fold change > 1.25 × up or down). *DAT*, *scb*, *CanA*-*14F*, *Nmdar1* and *CanB* genes were indicated in bold. The list was arranged by fold change and also included an Affymetrix DNA array probe set, gene symbol, FlyBase ID and Q-Value.
(XLS)

S2 Table Gene ontology (GO) terms in the biological process category present in differentially expressed genes between control (*w^1118^*) and *fmn* flies (*p* < 0.05). Count represents the number of differentially expressed genes belonging to indicated categories, respectively. *Nmdar1* gene was included in following 10 categories, localization, establishment of localization, transport, response to stimulus, neurological system process, system process, behavior, response to chemical stimulus, cell-cell signaling and transmission of nerve impulse, which were indicated in bold. The list was arranged by Count.
(XLS)
