# Supplementary figures and images for: The NMDA Receptor Promotes Sleep in the Fruit Fly, Drosophila melanogaster
Source: PLoS One. 2015 May 29;10(5):e0128101. doi: 10.1371/journal.pone.0128101 (PMC4449117; doi:10.1371/journal.pone.0128101)

S1 Fig.

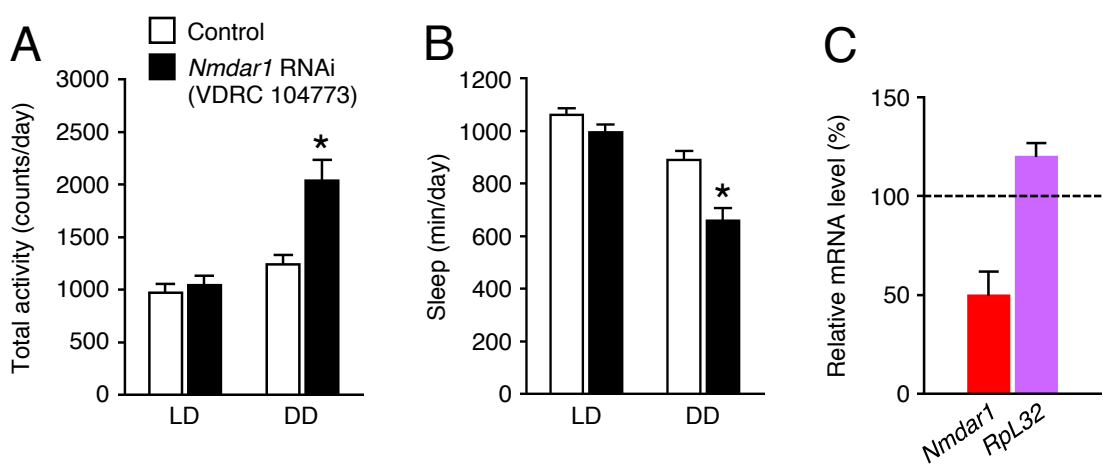

Supplement: S1 Fig — Total daily activity (A) and total sleep (B) for control (elav-Gal4;UAS-Dicer-2 × w 1118, white bars, n = 19) and Nmdar1 RNAi (VDRC 104773)-expressing flies using the elav-Gal4;UAS-Dicer-2 driver (black bars, n = 20) in LD and DD conditions. Data are presented as mean ± SEM. Asterisks indicate statistically significant differences compared to control according to a t-test (p < 0.05). (C) Efficiency of Nmdar1 gene knockdown. The expression levels of Nmdar1 and RpL32 genes in the head of male flies expressing Nmdar1 RNAi transgenes in all neurons are expressed as relative values to the control flies. Each mRNA level was quantified by qPCR and first normalized to GAPDH2. Then, the values were normalized to the average of independent control samples, which were set at 100%. Data are presented as mean ± SEM. n = 3 for each group. (PDF) [file pone.0128101.s001.pdf]

S2 Fig.

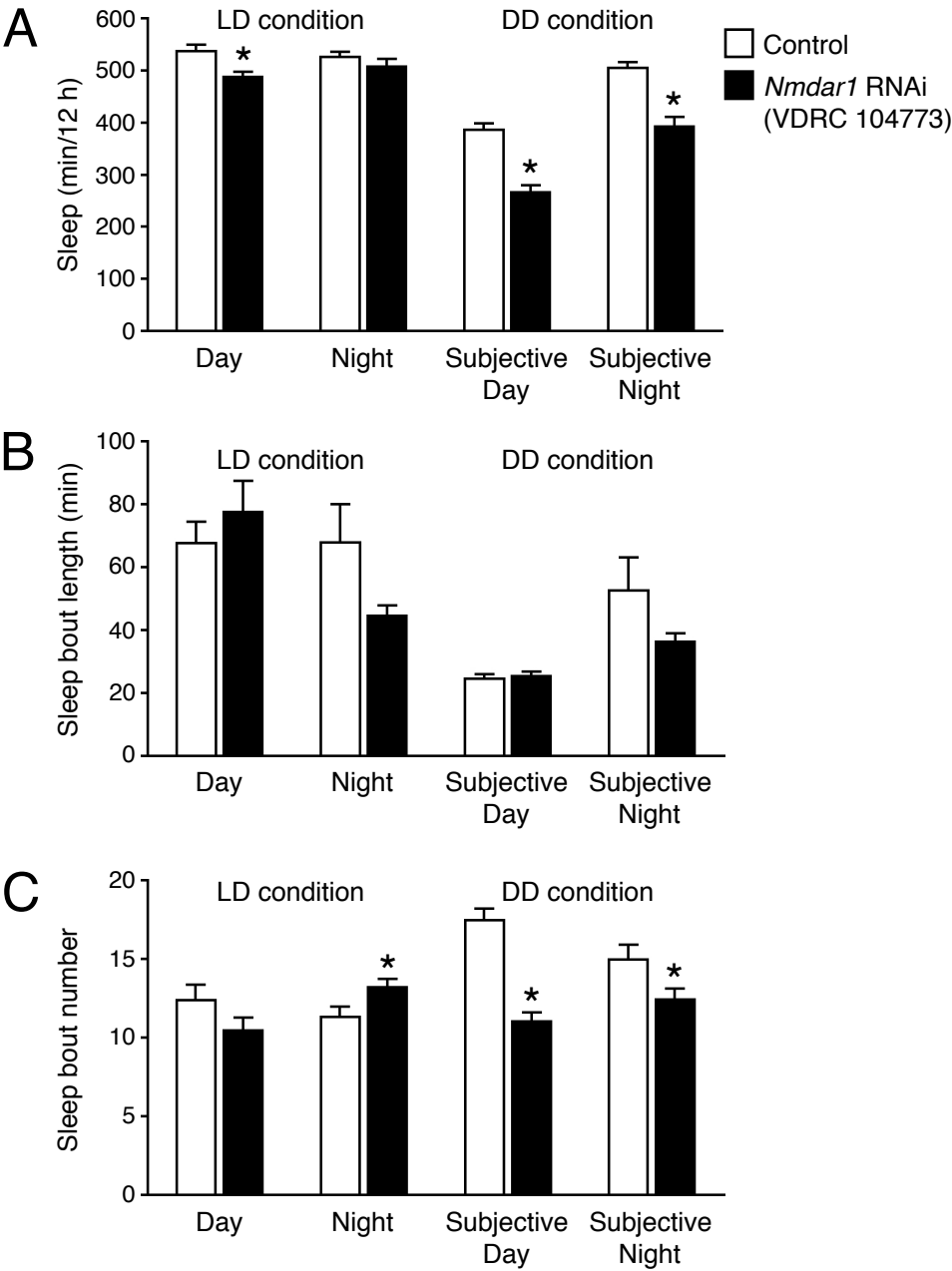

Supplement: S2 Fig — Total sleep (A), sleep bout length (B), and sleep bout number (C) for control (elav-Gal4;UAS-Dicer-2 × w 1118, white bars, n = 57) and Nmdar1 RNAi (VDRC 104773)-expressing flies using the elav-Gal4;UAS-Dicer-2 (black bars, n = 60) during day (ZT 0–12), night (ZT 12–24), subjective day (CT 0–12) and subjective night (CT 12–24). Data are presented as mean ± SEM. Asterisks indicate statistically significant differences from control determined by a t-test (p < 0.05). (PDF) [file pone.0128101.s002.pdf]

S3 Fig.

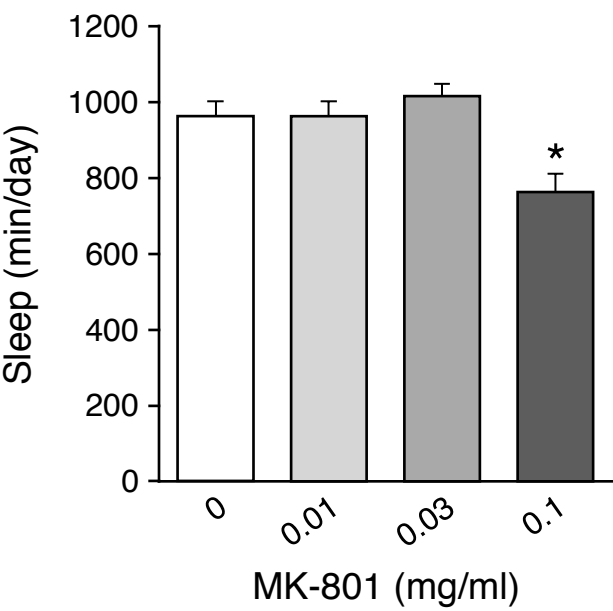

Supplement: S3 Fig — Total sleep for untreated control (white bars) and MK-801-fed w 1118 flies (colored bars) in DD conditions. MK-801 was directly mixed with sucrose-agar food at indicated concentration. Data are presented as mean ± SEM (n = 13–16 for each group). Groups with asterisks indicate statistically significant differences (Tukey-Kramer HSD test for normally distributed data, p < 0.05). (PDF) [file pone.0128101.s003.pdf]
